# Trends in neonicotinoid pesticide residues in food and water in the United States, 1999–2015

**DOI:** 10.1186/s12940-018-0441-7

**Published:** 2019-01-11

**Authors:** Hillary A. Craddock, Dina Huang, Paul C. Turner, Lesliam Quirós-Alcalá, Devon C. Payne-Sturges

**Affiliations:** 1 Maryland Institute for Applied Environmental Health, University of Maryland School of Public Health, 2234 L SPH, 255 Valley Drive, College Park, MD 20742 USA; 2 Department of Epidemiology and Biostatistics, University of Maryland School of Public Health, 255 Valley Drive, College Park, MD 20742 USA

**Keywords:** Neonicotinoids, Insecticides, Food, Water

## Abstract

**Background:**

Neonicotinoids are a class of systemic insecticides widely used on food crops globally. These pesticides may be found in “off-target” food items and persist in the environment. Despite the potential for extensive human exposure, there are limited studies regarding the prevalence of neonicotinoid residues in foods sold and consumed in the United States.

**Methods:**

Residue data for seven neonicotinoid pesticides collected between 1999 and 2015 by the US Department of Agriculture’s Pesticide Data Program (PDP) were collated and summarized by year across various food commodities, including fruit, vegetable, meat, dairy, grain, honey, and baby food, as well as water to qualitatively describe and examine trends in contamination frequency and residue concentrations.

**Results:**

The highest detection frequencies (DFs) for neonicotinoids by year on all commodities were generally below 20%. Average DFs over the entire study period, 1999–2015, for domestic and imported commodities were similar at 4.5%. For all the samples (both domestic and imported) imidacloprid was the neonicotinoid with the highest overall detection frequency at 12.0%. However, higher DFs were observed for specific food commodity-neonicotinoid combinations such as: cherries (45.9%), apples (29.5%), pears (24.1%) and strawberries (21.3%) for acetamiprid; and cauliflower (57.5%), celery (20.9%), cherries (26.3%), cilantro (30.6%), grapes (28.9%), collard greens (24.9%), kale (31.4%), lettuce (45.6%), potatoes (31.2%) and spinach (38.7%) for imidacloprid. Neonicotinoids were also detected in organic commodities, (DF < 6%). Individual commodities with at least 5% of samples testing positive for two or more neonicotinoids included apples, celery, and cherries. Generally, neonicotinoid residues on food commodities did not exceed US Environmental Protection Agency tolerance levels. Increases in detection trends for both finished and untreated water samples for imidacloprid were observed from 2004 to 2011.

**Conclusions:**

Analysis of PDP data indicates that low levels of neonicotinoids are present in commonly-consumed fruits and vegetables sold in the US. Trends in detection frequencies suggest an increase in use of acetamiprid, clothianidin and thiamethoxam as replacements for imidacloprid. Given these findings, more extensive surveillance of the food and water supply is warranted, as well as biomonitoring studies and assessment of cumulative daily intake in high risk groups, including pregnant women and infants.

**Electronic supplementary material:**

The online version of this article (10.1186/s12940-018-0441-7) contains supplementary material, which is available to authorized users.

## Introduction and background

Since their introduction in the 1990s, neonicotinoids have become the most widely used class of insecticide in the world, with registration in 120 countries [1–3]. In 2014, neonicotinoids represented more than 25% of the global pesticide market valued at over US $3 billion with thiamethoxam, imidacloprid and clothianidin accounting for almost 85% of the total neonicotinoid sales in crop protection in 2012 worldwide [4]. The leading developers of neonicotinoid pesticides include BayerCropScience, Mitsui Chemicals, Nippon Soda, Syngenta and Sumitomo [3].

Neonicotinoids are used for a large variety of agricultural production crops such as vegetables, pome and stone fruits, citrus, rice, cotton, corn, potato, sugar beet, oilseed rape, and soybean among other crops [1, 5]. In the United States, clothianidin (1850 US tons, with corn accounting for 95% in 2014) and imidacloprid (1000 US tons, with soybeans, vegetables and fruit accounting for 60% in 2014) are the most commonly used neonicotinoids in agriculture [6]. In addition to crop protection, application of neonicotinoids has expanded to home, lawn, and garden products to control termites, ants, cockroaches and turf pests, and flea and tick preventatives for dogs and cats [1, 7, 8]. Thus, multiple human exposure routes are plausible. [9–13]

Within 20 years of their introduction, neonicotinoids have rapidly replaced carbamate and organophosphate pesticides as a solution to human toxicity concerns and development of insect resistance from these older pesticide classes. Neonicotinoids possess several characteristics which make them attractive for use: predicted lower mammalian toxicity because of their selectivity for insect nicotinic acetylcholine receptors (nAChRs) over mammalian nAChRs; higher persistence; active against a broad spectrum of crop pests; systemic properties (e.g. transferring into all parts of treated plants, including pollen, nectar and guttation fluids, and the food produced by those plants [1, 14].); versatility in application (e.g. foliar sprays and prophylactic seed coating and soil treatment); high water solubility; and assumed lower impacts on fish and other wildlife [3, 11, 14, 15].

Over the last decade, however, neonicotinoids have been implicated in the colony collapse disorder of bees [16–18]. Three neonicotinoids, thiamethoxam, clothianidin and imidacloprid, were banned from use on flowering crops such as corn, oilseed rape and sunflowers in the European Union (EU) in 2013 for 2 years because of this unacceptable risk to bees [19]. In the spring of 2018, a majority of EU member states voted for a complete ban of these three neonicotinoids from all fields, except for use in closed greenhouses [20]. The ban is expected to go into effect by the end of 2018. Canada recently proposed tighter restrictions for clothianidin and thiamethoxam and is considering a ban on imidacloprid because of concerns that neonicotinoids are harmful to bees and are contaminating surface waters [21]. Several state and local jurisdictions in the United States have also recently enacted neonicotinoid bans, although the US Environmental Protection Agency (US EPA) registrations for approved uses of these pesticides remain in effect [22–24].

In addition to environmental issues, there is increasing concern about potential human exposures to neonicotinoids through the diet. Since 1999, the US government has tested food commodities for neonicotinoid residues [25, 26]. From 2010 to 2015, the Food and Drug Administration (FDA) also reported detecting neonicotinoids, including imidacloprid and acetamiprid, in several food commodities like fruits, vegetables, tea, and animal feed [25, 27]. For example, during fiscal year (FY) 2014 the FDA detected imidacloprid in 4 (1.1%) animal feed samples, 8 (38.1%) tea samples, and one (0.8%) honey sample. During the same year, the FDA detected imidacloprid in 27% of samples from their market basket survey [27]. A survey of US purchased food found 72% of fruits and 45% of vegetables had detectable neonicotinoids, including imidacloprid, acetamiprid, dinotefuran, flonicamid, thiacloprid, and thiamethoxam [28]. Recent surveys from the US, Belgium and Japan also report frequent food contamination from neonicotinoids, including acetamiprid, imidacloprid, thiacloprid, thiamethoxam, clothianidin, dinotefuran, and nitenpyram [12, 29, 30].

Acute and chronic exposures to neonicotinoids have resulted in measurable health effects on multiple organ systems in rodents, perhaps most notable on the neurological system and on developing pups in utero [31–36]. Emerging evidence raises concerns that neonicotinoids may also act as endocrine disruptors; testicular developmental effects of thiamethoxam have been reported in a 2-generational reproductive rat study and thiacloprid and imidacloprid may activate excess estrogen production in breast tissue by altering promoter activity [37, 38]. Both acetamiprid and imidacloprid exposure in utero is reported to adversely affect neuron development in rat brains. Researchers also reported that neonicotinoids could have similar effects as nicotine on the developing human brain [39–41]. This study contributed to the European Food Safety Agency’s (EFSA’s) 2013 decision to label neonicotinoids as potential developmental neurotoxicants and to establish an Acceptable Daily Intake (ADI) of 0.025 mg/kg/day for acetamiprid and 0.06 mg/kg/day for imidacloprid [42]. While epidemiological studies on neonicotinoids are limited [10], a few studies report modest associations between early life and in utero exposures and various conditions. Studies have reported that prenatal and adult exposures to neonicotinoids are linked with Autism Spectrum Disorder [43], heart defects [9], anencephaly [13], and neurological symptoms [44].

While the dietary route is believed to be a major route of human exposure to neonicotinoids [45], data to assess the potential extent of exposure via this route is limited. Obtaining country or state-specific data on annual neonicotinoid use on food commodities is difficult. Each state may report the data in different ways (e.g. overall quantities sold, applied or shipped), making comparisons challenging. While the US Geological Survey reports agricultural pesticide use based on farm surveys and estimates of harvested crop acres, these data are aggregated at the county level for broad commodity categories and do not address human dietary exposures. To help address this knowledge gap, we describe trends in neonicotinoid residues on fruit, vegetables, meat, and dairy sold in the US (both domestic and imported commodities) as well as water from 1999 to 2015 using publically available data from the US Department of Agriculture (USDA) Pesticide Data Program (PDP). Results from our analysis will help identify potential sources of neonicotinoid exposures which, in light of the potential human health effects, is critical for driving future policy and research.

## Methods

### Neonicotinoid residue data from the pesticide data program

The USDA’s PDP collects annual data on pesticide residues on a wide variety of fresh, processed domestic, imported, organic and conventional food commodities sold in the US, including foods most likely consumed by infants and children and, to the extent possible, “as eaten” (i.e., samples that are prepared emulating consumer practices) [26]. The PDP commodity sampling protocol is based on a rigorous statistical study design to ensure data are representative of the US food supply [26]. Commodity samples tested by the PDP are chosen at random, close to the point of consumption (i.e., terminal markets and large chain store distribution centers from which food commodities are supplied to supermarkets and grocery stores), from 11 states to represent about 50% of the nation’s population and all four census regions of the United States [26]. Sampling sites are selected to represent major US producers of fruits and vegetables [26]. Further detailed description of sampling and analytical methods used in the PDP are available on USDA’s web site [26]. Since 1991, the PDP has tested more than 95 different commodities, including fruits, vegetables, juices, meats, dairy and grains and prepared baby foods and water samples from bottled water, groundwater from municipal systems, private residences, schools, and daycare facilities (2007 through 2013), and finished and untreated water from municipal systems (2001–2013) [26]. We obtained all available neonicotinoid residue data from the USDA’s PDP from 1999 through 2015. USDA began monitoring for imidacloprid in 1999 and included additional neonicotinoids over subsequent years [46]. Table 1 shows the year in which PDP sampling for specific neonicotinoids began, total number of commodities sampled since 1999 and the respective US EPA reference doses (RfDs) as a measure of toxicity. Not every commodity-neonicotinoid combination is sampled every year and PDP’s water surveys for raw/finished drinking water and groundwater were discontinued in 2013 due to resource limitations. Thus, we limited our analysis to commodities for which PDP data were available.

**Table 1:** Year in which PDP sampling for each individual neonicotinoid was initiated

Year Sampling Began	Neonicotinoid	Total number of commodities sampled through 2015	US EPA Reference Dose^a^ mg/kg/day
1999	Imidacloprid	131,369	0.06
2003	Thiamethoxam	113,368	0.006
2004	Clothianidin^b^	92,818	0.01
2005	Dinotefuran	78,027	0.02
2005	Thiacloprid	62,960	0.004
2004	Acetamiprid	98,535	0.07
2008	Flonicamid	68,903	0.04

^a^The maximum daily oral dose of the neonicotinoid estimated to be without an appreciable risk of harm over a lifetime

^b^Additionally a metabolite of Thiamethoxam

### Data analysis

We conducted our analysis on residue levels for seven neonicotinoid pesticides (acetamiprid, clothianidin, dinotefuran, flonicamid, imidacloprid, thiacloprid and thiamethoxam) included in the PDP (Table 1). Data on imidacloprid urea was excluded from our analysis since it is a breakdown product of imidacloprid. To accommodate the temporal discontinuity in the PDP, we combined the data into two classifications of commodities, presented as “Commodity Hybrid” and “Major Commodity” (see Additional file 1). “Commodity Hybrid” combines individual commodities up one level of specificity from the USDA commodity codes. For example, apples-single servings, apple juice and applesauce were combined into one Commodity Hybrid classification of “apple”. “Major Commodity” presents the data with the least resolution, but is useful in broadly examining trends in residues to which people may be exposed through their diet. For Major Commodity we used six food commodities: fruit, vegetable, meat, dairy (e.g. milk, butter, cream, yogurt), grain (including rice) and nuts. The PDP also provides information on country of origin, which allowed us to assign either domestic or import designations to every neonicotinoid-commodity combination and to estimate import fraction of each commodity. Commodities labeled as organic are also included in the PDP. We used SAS 9.4 (SAS Institute, Cary NC) to perform the analyses. We calculated the detection frequencies based on limits of detection (LOD); LODs varied by year for individual neonicotinoids (see Additional file 2). We estimated detection frequency for neonicotinoids by origin (imported and domestic) and for each specific neonicotinoid-Commodity Hybrid and Major Commodity combination by year using the PROC FREQ procedure. Organic and conventionally grown food commodities were combined in our main analysis since we expected residues detection among organics to be low. We also separately evaluated commodities labeled as organic and individually identified as baby food items, water samples, and honey. For estimating mean residue levels, PROC MEANS was used and we only evaluated residue levels above the limit of detection (LOD). Figures were generated using Microsoft Excel. Because LODs for neonicotinoids varied widely across the sampling period, as part of our sensitivity analyses, we assessed temporal trends of detection for the most frequently detected neonicotinoid (imidacloprid) among fruits and vegetables by setting the LODs to the highest LOD reported for these commodities during the sampling period. PROC FREQ procedure was used.

## Results

In the present study, we describe trends in neonicotinoid residues across various fruit, vegetable, meat, dairy, water, grain, honey and baby food commodities as reported by the USDA PDP for the years 1999–2015. Over this time period, the PDP tested over 645,980 samples in 103 different individual food commodities.

### Individual commodity neonicotinoid residue trends

Neonicotinoids were mainly detected in fruits and vegetables (DF < 20%), and less frequently in other major commodity groups. Therefore, we examined neonicotinoid concentrations by individual commodity groups (Commodity Hybrid) among all domestic and imported fruits and vegetables for the period 1999–2015. Specific commodity groups where neonicotinoids were detected on at least 20% of the samples included: Acetamiprid for apple commodities (32.5%), cherries (45.9%) pears (24.3%) and strawberries (21.3%); and imidacloprid in cauliflower (57.5%), celery (20.9%), cherries (26.3%), cilantro (30.6%), grapes (28.9%), greens-collard (24.9%), greens-kale (31.4%) and lettuce (45.6%), potatoes (31.2%) and spinach (38.7%) (See Additional file 3). However, among commodities with lower detection frequencies, we found some of the highest maximum residue levels for acetamiprid in green/leafy vegetables, including collard greens (1.4 ppm), kale (1.6 ppm) and organic spinach (1.6 ppm, which is in violation of USDA organic standard); flonicamid in lettuce (1.2 ppm) and spinach (3.8 ppm); imidacloprid in organic broccoli (1.5 ppm, which is in violation of USDA organic standard), cilantro (1.1 ppm), grapes (2.3 ppm), kale (1.0 ppm) and spinach (1.0 ppm); and dinotefuran in cherry tomatoes (3.0 ppm).

We also found that different neonicotinoids were used on the same commodity types. For example, all seven neonicotinoids were detected on pepper samples with average concentrations of 0.015 ppm, 0.013 ppm, 0.036 ppm, 0.027 ppm, 0.054 ppm, 0.022 ppm, and 0.014 ppm for acetamiprid, clothianidin, dinotefuran, flonicamid, imidacloprid, thiacloprid and thiamethoxam, respectively. Six neonicotinoids were detected on cauliflower, celery, kale, strawberries, summer squash and watermelon. We also note that multiple neonicotinoids were detected on single commodity samples. Commodities with at least 5% of individual samples testing positive for two or more neonicotinoids included apples, celery, cherries, peppers, spinach, strawberries, summer squash and tomatoes.

In general, mean neonicotinoid residue concentrations reported for all commodities did not exceed US EPA tolerance levels (i.e., the maximum amount of a pesticide residue that is legally allowed to remain on or in a conventionally grown food) [47]. However, several commodity group samples had reported maximum residues during 1999–2015 which approached (> 50% of the tolerance level) or exceeded US EPA established tolerance levels as shown in Additional file 3. For example, maximum residue concentrations on strawberries and green beans exceeded US EPA tolerance levels for acetamiprid and dinotefuran, respectively. Additionally, maximum reported residue concentrations on tomatoes exceeded EPA tolerance levels for dinotefuran and flonicamid, while maximum residue concentrations reported for pears exceeded the US EPA tolerance levels for thiacloprid. Lastly, maximum thiamethoxam residues on cucumber and strawberries were 95 and 83% of US EPA tolerance, respectively, and maximum thiamethoxam residues reported for tomatoes exceeded tolerance levels.

### Major commodity neonicotinoid residue trends

Table 2 shows summary results of neonicotinoid residues detected in all samples analyzed by the PDP grouped by major commodity type (Major Commodity) over the period 1999–2015. Specific commodities with maximum concentrations are noted along with the year and country of origin. As aforementioned, neonicotinoids were mainly detected in fruits and vegetables (DF ≤ 20%), and less frequently in other major commodity groups. The highest overall detection frequency was for imidacloprid (20%) among vegetables and acetamiprid (13%) among fruits. Of all 15,410 dairy samples tested, one domestic butter sample tested positive for imidacloprid, in 2012 (0.0053 ppm). Samples of beef products, poultry products (including eggs), pork products, fish and honey in the PDP 1999–2015 all tested below the LOD. We also evaluated major commodity level trends over this same time period for domestic and imported items separately; these results are shown in Additional file 4.

**Table 2:** Summary of Neonicotinoid Concentrations by Major Commodity,1999–2015

Neonicotinoid	Major Commod	Total N	N > LOD	Detect Freq %	Mean Conc^a^ (ppm)	Min Conc (ppm)	Max Conc (ppm)	Commodity with max conc (year, domestic or import and country of origin)
Acetamiprid	Fruits	33,728	4514	13.38	0.0043	0.001	1.5	Raspberries(2013, domestic)
	Vegetables	49,987	1607	3.21	0.0011	0.001	1.6	Greens, Kale(2007, domestic); Spinach(2009, domestic, organic)
	Meat	5468	0	0	0	0	0	
	Dairy	4480	0	0	0	0	0	
	Grain (includes Rice)	2580	0	0	0	0	0	
	Nuts	315	0	0	0	0	0	
Clothianidin	Fruits	34,721	333	0.96	0.0004	0.0013	0.51	Grapes(2015, domestic)
	Vegetables	43,992	1094	2.49	0.0005	0.0025	0.38	Spinach(2015, domestic)
	Meat	3454	0	0	0	0	0	
	Dairy	2203	0	0	0	0	0	
	Grain (includes Rice)	6384	1	0.02	0	0.0028	0.0028	Sweet Corn, Frozen(2014, domestic)
	Nuts	315	0	0	0	0	0	
Dinotefuran	Fruits	28,783	187	0.65	0.0003	0.004	0.35	Grapes(2009, domestic)
	Vegetables	40,198	708	1.76	0.0009	0.004	3.00	Cherry Tomatoes(2012, domestic)
	Meat	2920	0	0	0	0	0	
	Dairy	2996	0	0	0	0	0	
	Grain (includes Rice)	2580	3	0.12	0	0.037	0.049	Rice(2014, domestic, organic)
	Nuts	315	0	0	0	0	0	
Flonicamid	Fruits	26,455	271	1.02	0.0010	0.0017	0.6	Strawberries(2015, domestic)
	Vegetables	35,607	1093	3.07	0.0054	0.0017	3.8	Spinach(2008, domestic)
	Meat	1201	0	0	0	0	0	
	Dairy	3688	0	0	0	0	0	
	Grain (includes Rice)	1952	0	0	0	0	0	
	Nuts	0	0	0	0	0	0	
Imidacloprid	Fruits	50,097	3952	7.89	0.0036	0.0002	2.3	Grapes(2010, import from Chile)
	Vegetables	55,278	10,999	19.9	0.0036	0.0002	1.5	Broccoli(2013, domestic, organic)
	Meat	5498	0	0	0	0	0	
	Dairy	4480	1	0.02	0	0.0053	0.0053	Butter(2012, domestic)
	Grain (includes Rice)	8146	1	0.01	0	0.0002	0.011	Rice(2009, import from India)
	Nuts	862	0	0	0	0	0	
Thiacloprid	Fruits	29,707	905	3.05	0.0006	0.0007	0.3385	Pears(2015, import from Chile)
	Vegetables	26,078	60	0.23	0.0001	0.0007	0.49	Snap Peas (2012, import from Guatemala)
	Meat	3367	0	0	0	0	0	
	Dairy	2204	0	0	0	0	0	
	Grain (includes Rice)	1604	0	0	0	0	0	
	Nuts	0	0	0	0	0	0	
Thiamethoxam	Fruits	39,367	730	1.85	0.0004	0.0013	0.28	Tangerines (2012, import from South Africa)
	Vegetables	56,740	2082	3.67	0.0006	0.002	0.38	Cherry Tomatoes(2012, import from Mexico)
	Meat	3836	0	0	0	0	0	
	Dairy	3738	0	0	0	0	0	
	Grain (includes Rice)	7395	1	0.01	0	0.0025	0.0025	Sweet Corn, Fresh(2010, import from Mexico)
	Nuts	315	0	0	0	0	0	

^a^Just among samples above LOD; See Additional file 2 for LODs by year, commodity and neonicotinoid

Additionally, we assessed neonicotinoids among all organic commodities. Table 3 provides a summary of neonicotinoid residue detection by major commodity type. Nearly 3% of organic fruits were found to have detectable acetamiprid and thiacloprid residues. Organic domestic spinach, rice and broccoli samples were identified as specific commodities with maximum residue levels during 1999–2015; maximum residue levels for these commodities exceeded those reported in conventionally grown samples of the same commodities. Still, overall average DF of neonicotinoids among all organic commodity samples was very low at < 1%.

**Table 3:** Summary of Neonicotinoid Concentrations by Organic Major Commodity,1999–2015

Neonicotinoid	MajorCommod	Total N	N > LOD	Detect Freq %	Mean Conc^a^ (ppm)	Min Conc (ppm)	Max Conc (ppm)	Organic commodity with max conc (year, domestic or import and country of origin)
Acetamiprid	Fruits	1175	31	2.64	0.016	0.002	0.058	Cherries, frozen; imported 2015 Turkey
	Vegetables	2003	13	0.65	0.14	0.0017	1.60	^b^Spinach; domestic 2009
	Meat	23	0	0	0	0	0	
	Dairy	310	0	0	0	0	0	
	Grain (includes Rice)	64	0	0	0	0	0	
	Nuts	11	0	0	0	0	0	
Clothianidin	Fruits	1087	3	0.28	0.055	0.015	0.12	Grapes; domestic 2010
	Vegetables	1963	5	0.25	0.071	0.003	0.21	Sweet Bell Peppers; imported 2012 Mexico
	Meat	23	0	0	0	0	0	
	Dairy	134	0	0	0	0	0	
	Grain (includes Rice)	65	0	0	0	0	0	
	Nuts	11	0	0	0	0	0	
Dinotefuran	Fruits	994	0	0	0	0	0	
	Vegetables	1264	5	0.40	0.045	0.01	0.18	Cherry Tomatoes; imported 2012 Dominican Republic
	Meat	23	0	0	0	0	0	
	Dairy	242	0	0	0	0	0	
	Grain (includes Rice)	64	1	1.56	0.049	0.049	0.049	^b^Rice; domestic 2014
	Nuts	11	0	0	0	0	0	
Flonicamid	Fruits	1024	0	0	0	0	0	
	Vegetables	1681	16	0.95	0.178	0.002	1.50	Spinach; domestic 2015
	Meat	0	0	0	0	0	0	
	Dairy	257	0	0	0	0	0	
	Grain (includes Rice)	21	0	0	0	0	0	
	Nuts	0	0	0	0	0	0	
Imidacloprid	Fruits	1383	13	0.94	0.027	0.002	0.095	Cherries; domestic 2007
	Vegetables	2102	31	1.47	0.062	0.0002	1.50	^b^Broccoli; domestic 2013
	Meat	23	0	0	0	0	0	
	Dairy	310	0	0	0	0	0	
	Grain (includes Rice)	111	0	0	0	0	0	
	Nuts	23	0	0	0	0	0	
Thiacloprid	Fruits	1054	27	2.56	0.043	0.006	0.098	Cherries, frozen; imported 2015 Turkey
	Vegetables	1224	0	0	0	0	0	
	Meat	23	0	0	0	0	0	
	Dairy	189	0	0	0	0	0	
	Grain (includes Rice)	15	0	0	0	0	0	
	Nuts	0	0	0	0	0	0	
Thiamethoxam	Fruits	1258	3	0.24	0.001	0.0020	0.024	Strawberries; domestic 2015
	Vegetables	2180	6	0.28	0.018	0.0025	0.078	Sweet Bell Peppers; imported 2012 Mexico
	Meat	23	0	0	0	0	0	
	Dairy	264	0	0	0	0	0	
	Grain (includes Rice)	65	0	0	0	0	0	
	Nuts	11	0	0	0	0	0	

^a^Just among samples above LOD; See Additional file 2 for LODs by year, commodity and neonicotinoid

^b^Maximum residue concentration among all major commodities of same type

### Neonicotinoid residues in samples of baby food and infant formula

Among the baby food and infant formula samples, peach, pear, and applesauce had detectable levels of acetamiprid, imidacloprid and thiacloprid (see Table 4). Similar to overall results described above, some of these commodities tested positive for multiple neonicotinoids. Thirteen percent (*N* = 744) of apple sauce and 5 % of pear (*N* = 776) samples had two or more neonicotinoids detected. No baby foods tested had neonicotinoid residue concentrations above the LOD for clothianidin (*N* = 3953), dinotefuran (*N* = 3916), flonicamid (*N* = 3740), or thiamethoxam (*N* = 5257). Additionally, no samples of dairy-based infant formula (*N* = 4935), baby food carrots (*N* = 3168), peas (*N* = 3092), green beans (*N* = 5432), sweet potatoes (N = 5432) or soy-based infant formula (*N* = 4942) tested above the LOD.

**Table 4:** Summary of Neonicotinoid Concentrations by Baby Food Type, 1999 - 2015

USDA Commod Code	Baby Food	Acetamiprid					Imidacloprid					Thiacloprid					% of samples with 2 or more neonics	% of samples with 3 or more neonics
		N	DF%	^a^Mean Conc (ppm)	^a^Min Conc (ppm)	^a^Max Conc (ppm)	N	DF %	^a^Mean Conc (ppm)	^a^Min Conc (ppm)	^a^Max Conc (ppm)	N	DF%	^a^Mean Conc (ppm)	^a^Min Conc (ppm)	^a^Max Conc (ppm)		
AC	Apple-sauce	744	51.5	0.005	0.001	0.031	744	17.5	0.000	0.000	0.003	744	12.8	0.00	0.00	0.01	13.17	1.34
IH	Peaches	777	35.9	0.003	0.002	0.009	777	0.0	0.000	0.000	0.000	777	0.0	0.00	0.00	0.00	0.00	0.00
IP	Pears	776	25.0	0.012	0.002	0.046	776	10.1	0.007	0.002	0.025	776	10.8	0.01	0.00	0.03	5.28	6.44

*DF* detection frequency

No other neonicotinoids were identified in baby or infant foods

These data also contributed to the overall commodity findings

^a^mean concentrations and range of concentrations were calculated among commodities with pesticide detected; See Additional file 2 for LODs by year, commodity and neonicotinoid

### Temporal trends in proportion of neonicotinoid samples testing above the limits of detection (LOD)

**Fig. 1:**
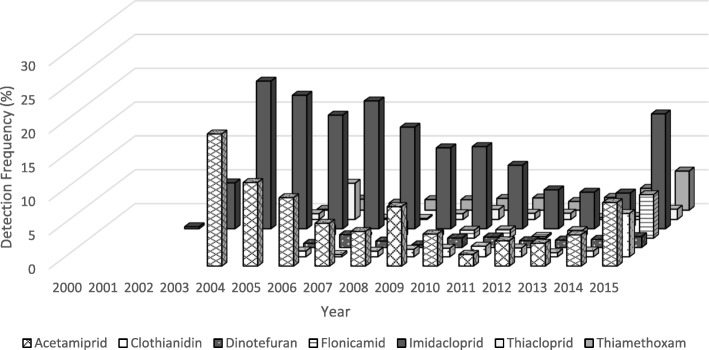
Proportion of domestic samples from the PDP above the LOD among all neonicotinoids, by year and neonicotinoid type, 1999–2015. Please see Additional file 2 for LODs by year, commodity and neonicotinoid

**Fig. 2:**
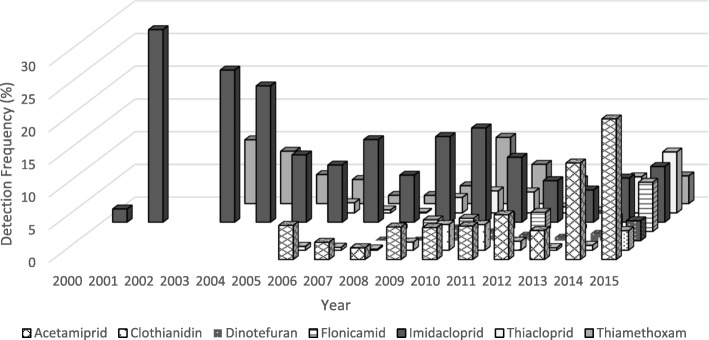
Proportion of import samples from the PDP above the LOD among all neonicotinoids, by year and neonicotinoid type, 1999–2015. Please see Additional file 2 for LODs by year, commodity and neonicotinoid

The number of commodities tested for neonicotinoids by PDP has generally increased over the years, however there was a large drop in the number of samples tested for imidacloprid from 2014 to 2015 (see Additional file 5). Figures. 1 and 2 detail the detection frequencies (DF) for all seven neonicotinoids by year and origin (i.e., domestic or import). Neonicotinoid detection frequencies across all domestic commodities were below 15%, except for imidacloprid (DFs =15–20%) during the years 2004–2007, and 2015 (Fig. 1). For all imported commodities (Fig. 2), detection frequencies for neonicotinoids were below 20% for most of the years reported, except for imidacloprid in 2001, 2003 and 2004. For all commodity samples, both domestic and imported, imidacloprid was the neonicotinoid with the highest overall detection frequency at 12% (reaching as high as 56% for certain commodities in specific years) from 2000 to 2015. Only 202 samples were tested in 1999 and no neonicotinoids were detected in these samples. As shown in Fig. 1, the proportion of domestic samples above the LOD for all neonicotinoids peaked from 2005 to 2008, and generally decreased until 2013, when detection frequencies started to increase (2014–2015), particularly for acetamiprid, clothianidin, flonicamid, imidacloprid and thiamethoxam. The number of imported commodities testing positive for neonicotinoid residues decreased from 2001 to 2009 but then slight increase in 2010 and 2011 followed by a downward trend in subsequent years. However, in 2015 we observed an increase in detection frequencies for all neonicotinoids among imported commodity samples.

**Fig. 3:**
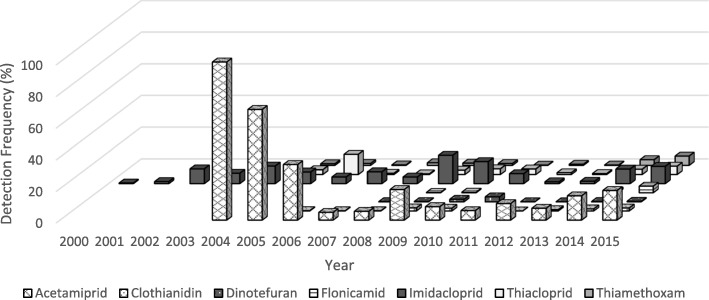
Proportion of all fruit (major commodity) samples from the PDP above the LOD among all neonicotinoids, by year and neonicotinoid type, 1999–2015. Please see Additional file 2 for LODs by year, commodity and neonicotinoid

**Fig. 4:**
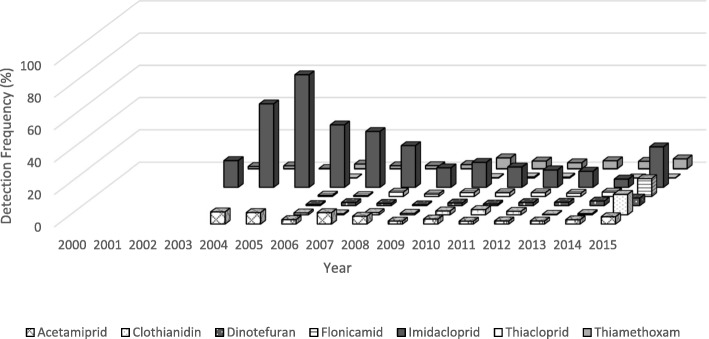
Proportion of all vegetable (major commodity) samples from the PDP above the LOD among all neonicotinoids, by year and neonicotinoid type, 1999–2015. Please see Additional file 2 for LODs by year, commodity and neonicotinoid

**Fig. 5:**
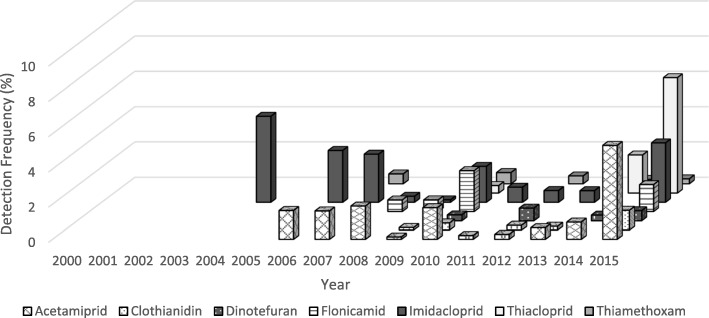
Proportion of organic samples from the PDP above the LOD among all neonicotinoids, by year and neonicotinoid type, 1999–2015. Please see Additional file 2 for LODs by year, commodity and neonicotinoid

Trends in detection frequencies among fruit, vegetable, and all organic commodities are displayed in Figs. 3, 4 and 5. We observed a similar decreasing trend in neonicotinoid DFs for fruits and vegetables from 2004 to 2006 through 2014 when detection frequencies started to increase. Acetamiprid had the highest DF at 100% in 2004 and 70% in 2005 among fruits, whereas imidacloprid had the highest DF at 70% in 2005 among vegetables. Neonicotinoids were also detected in organic commodities, although at lower rates than those observed for all commodities. However, DFs of neonicotinoid residues in organic commodities also increased in 2015, specifically for thiacloprid (DF = 7%), imidacloprid (DF = 3%) and acetamiprid (DF = 5%).

In our sensitivity analysis (Additional file 6), when setting the LOD to the highest LOD reported, we observed a similar temporal trend of detection frequency as was observed when using individual varying LODs for fruits; DFs peaked around 2009–2010 for fruits and then DFs decrease until 2015 when DF increased. For vegetables, the DFs observed using individual varying LODs was consistently high throughout the sampling years. The DFs peaked around 2015 and then gradually decreased until 2015. Conversely, the DFs using the highest LOD for vegetables reported were consistently low with a peak in 2008.

### Neonicotinoid residues in water samples

Untreated and treated water samples were tested for acetamiprid, clothianidin, imidacloprid and thiamethoxam with concentrations ranging from 8 ppt to 202 ppt. We observed increasing detection trends for both finished and untreated water samples for imidacloprid from 2004 to 2011 (see Additional file 7). The highest detection frequency for imidacloprid in untreated and treated water was 36.7 and 29.7% in 2011, respectively, though samples collected in 2012 showed lower detection frequencies. Of all the neonicotinoids, only imidacloprid was detected in the samples; the average concentration among positive groundwater samples ranged from 0.24 ppt to 0.33 ppt from 2010 to 2013.

## Discussion

In this study, we report detection of neonicotinoids in domestic and imported fruits and vegetables sold in the US as well as in water samples from 1999 to 2015. The most commonly detected neonicotinoids were imidacloprid and acetamiprid. However, we found a decreasing trend in detection frequency for imidacloprid from an overall peak of ~ 20% during 2004–2007 to less than 5% in 2014 among domestic commodities. This peak detection appears to coincide with an increase in use of imidacloprid worldwide during this same period [1, 3]. We also observed a decline in detection of imidacloprid on imported commodities; however, detection frequencies were generally similar or lower than domestic commodities. After 2014, we observed increases in detection of acetamiprid, clothianidin and thiamethoxam and flonicamid in domestic and all neonicotinoids among imported commodities. Pesticide sales and use data indicate that clothianidin and thiamethoxam are replacing imidacloprid even though both are potentially more toxic than imidacloprid [3, 6, 48–50]. It has been suggested that growers may be switching to these and other neonicotinoids because of increasing pest resistance to imidacloprid [1].

Neonicotinoids were also detected in organic commodities. The temporal trends in DFs among organic commodities were similar to those observed for all commodities; after peak detection in 2004–2007, detection frequencies decreased until 2015 when they increased. While the overall detection rate of any neonicotinoid among all organic commodities over the study time period was < 1%, which is considerably lower compared to all commodities we analyzed in the PDP, we observed an increase in detection rates in 2015 for thiacloprid, acetamiprid and imidacloprid to 6, 2 and 2%, respectively. Three domestic organic commodity samples were identified as commodities with maximum residue levels (spinach 1.6 ppm acetamiprid; rice 0.049 ppm dinotefuran; and broccoli 1.50 ppm imidacloprid); residue levels in these samples were higher than those reported for the same conventional major commodity category. The organic spinach and broccoli samples violate the USDA organic standard for these neonicotinoid residues. Although USDA organic standards prohibit the use of most synthetic pesticides for at least 3 years prior to the harvest of an organic crop, pesticide residues have been previously detected in organic commodities [51]. A recent pilot study conducted by USDA found that 42.7% of 571 samples of organic apples, bell peppers, potatoes, strawberries and tomatoes had detectable pesticide residues [52]. Authors reported that imidacloprid was detected on organic tomato samples, albeit at allowable maximum residue tolerance levels for organic produce (< 5% of the US EPA tolerance level) [52]. Possible factors that account for pesticide residues in organic samples include product mislabeling; misidentification of the samples by the PDP; post harvest contamination; inadvertent, unavoidable contamination from environmentally persistent pesticides; or drift from pesticides applied to adjacent land [52, 53]. Given the physical characteristics of neonicotinoids (e.g., systemic, high water solubility, and persistence in soils [3, 54]), better procedures may be needed to limit or prevent organic crops from being grown in soils contaminated with neonicotinoids. Additional safeguards may be warranted to ensure that farmers are fully aware if the seeds they are purchasing have been treated with neonicotinoids [54, 55].

Documenting the patterns of neonicotinoid residues in individual food commodities is an important step towards understanding potential dietary human exposures and helps in identifying specific commodities that may be of potential concern based on observed residue levels. Due to the discontinuity of the data from 2009 to 2015, we aggregated data from multiple years (2009–2015) and focused on the detection frequency by types of commodities. Neonicotinoids were detected most frequently (21–58%) in apples, cauliflower, celery, cherries, cilantro, grapes, collard greens, kale, lettuce, pears, potatoes, spinach and strawberries. Residues of multiple neonicotinoids were detected on samples of several commodity types such as cherries and strawberries. While the majority of commodities had neonicotinoid residue levels well below legal tolerance levels established by the US EPA, some commodities had reported maximum residue levels approaching or exceeding these limits such as tomatoes (acetamiprid and flonicamid), green beans (clothianidin and dinotefuran), strawberries (acetamiprid, imidacloprid and thiamethoxam) and grapes (clothianidin and imidacloprid). Commodities highlighted in the present study are also among the fruits and vegetables commonly consumed by young children under 5 years of age [56]. Additionally, neonicotinoids were detected in prepared baby foods predominantly peach, pear, and applesauce. Further, the presence of multiple neonicotinoids on single commodity samples raises concerns about cumulative exposures and risks. US EPA has not conducted a human health cumulative risk assessment for neonicotinoids per requirements under Food Quality Protection Act (FQPA) for determining tolerance levels for these pesticides [57]. Concern for cumulative exposures and risks is further heightened by the fact that in addition to neonicotinoids, multiple classes of pesticides (e.g. organophosphate pesticides) are regularly used on fruits and vegetables [26]. Health risks from exposures to mixtures of multiple pesticide residues may be higher than currently estimated; however, further research is needed to understand potential additive or synergistic effects of cumulative exposures to pesticides.

Our study supports findings from prior smaller scale studies of neonicotinoid residues in US foods [28, 30, 45]. We report detection of these pesticides in fruits and vegetables, but over a longer period of time and over a larger number of different commodities than previously reported. Similar to prior studies [28, 30, 45], imidacloprid and thiamethoxam were the most frequently detected neonicotinoids. However, neonicotinoid detection frequencies in the PDP survey were consistently lower than those reported previously. This is likely due to the more sensitive analytical methods with lower limits of detection used in these prior independent studies [28, 30, 45]. For example, USDA PDP detection limits for imidacloprid ranged from 0.001 to 0.019 ppm, while Chen et al. reported LODs ranging between 0.0001 and 0.0005 ppm. The PDP tested many commodity samples from 1999 to 2015 for neonicotinoids (*N* > 645,980), while Chen et al. (2014) only tested 29 fruit and vegetable samples from grocery stores in Boston and the Lu et al. study (2018) analyzed 64 fruit and vegetable samples from US Congressional Cafeterias in 2015. The differences in sample size and limits of detection make comparisons across studies, including ours with PDP data, challenging.

Zero detection of neonicotinoids in PDP honey samples from 1999 to 2015 in our analysis stands in stark contrast with small studies led by others [28, 58]. Given the systemic properties of neonicotinoids, these pesticides can penetrate and translocate through the plant including pollen and nectar [14, 54, 59]. Bees rely on nectar and pollen for energy and use nectar from flowers to make honey. A study by Chen et al., (2014) identified neonicotinoids in 90% of honey samples tested, whereas the PDP did not identify neonicotinoids in any honey samples. A recent global survey of neonicotinoid contamination in honey samples found 75% of all 198 samples had at least one of the 5 neonicotinoids tested and 45% contained two or more of these pesticides [58]. Results varied by region. In North America, 86% of samples had detectable levels of neonicotinoids [58]. In Europe, where a partial ban was in place at the time, neonicotinoids were detected in 79% of the samples tested [58]. In response to growing interest on the impact of neonicotinoids on bees, USDA PDP program conducted a separate honey survey in 2017. Among the 315 honey samples collected, no neonicotinoids were detected. This discrepancy could be related to the sensitivity of the analytical methods used.

The presence of neonicotinoids in drinking water supplies and in the one dairy sample provide evidence of the potential for these pesticides to be found in “off-target” food items and to persist in the environment. USDA began pesticide monitoring of raw intake and finished drinking water in 2001 and ended the program in 2013 due to financial constraints. Water samples were taken from close to 100 sites among 30 states. In our analysis, we observed an increasing trend in detection frequency of imidacloprid from approximately 2 % in 2004 to 36.7% in 2011 for untreated water and < 1% in 2002 to 29.7% in 2011 for finished drinking water. After 2011, the detection frequencies dropped to around 5–7% for both types of water. These declines could be due to variation in the timing and location of water samples taken by USDA across this time period, changes in pesticide use or difference in LODs of analytical methods used.

Neonicotinoids are commonly used in agriculture and surveys of streams in farming-intensive regions in the US have found that neonicotinoid residues are widespread in surface waters [60, 61]. It has also been noted in other studies that neonicotinoid levels in surface water spike around planting season, suggesting that seeds treated with neonicotinoids may pose more of a threat to surface water quality than other neonicotinoid preparations [60]. Additionally, the US Geological Survey found widespread neonicotinoid contamination in urban streams [60], suggesting that urban uses of neonicotinoids on lawns, gardens, and recreational public spaces can have a measurable impact on watershed contamination, including sources of tap water. It also appears from the PDP data that drinking water treatment techniques may not be effective in removing imidacloprid from intake waters as concentrations of imidacloprid were similar for untreated and finished water. A recent study in Iowa reported that neonicotinoids can persist during water treatment and distribution, and upon testing drinking water from the treatment system detected clothianidin (3890–57,300 ppt), imidacloprid (1220–39,500 ppt), and thiamethoxam (240–4150 ppt) in 100% of samples they collected. In the PDP water samples, only imidacloprid was detected, it was not detected in all samples (e.g. the highest DF was 36.7%), the concentrations were much lower, ranging from 8 ppt to 202 ppt [57]. This significant difference in the findings may be due to the large amount of corn and soybeans, among many other agricultural crops, grown in Iowa. This study also suggests that granular activated carbon filtration may be a more effective treatment technique for decreasing neonicotinoid concentrations in finished drinking water than conventional sand filtration [62]. To date, EPA has not established a mandatory drinking water limit (Maximum Contaminant Level, MCL) for any neonicotinoid pesticide.

Our results should be interpreted with caution due to some study limitations. First, commodities sampled by the PDP varied from year to year and therefore specific neonicotinoid-commodity combinations are not measured for each year of the study. The PDP tests about 10 or 12 different food commodities a year, typically testing about 500 to 700 samples of each food commodity. The foods selected rotate from 1 year to the next and only a limited number of foods have been tested for as many as three consecutive years. The number of foods analyzed for neonicotinoids varied over our study time period, with the USDA gradually increasing the number of food items since 1999 (*n* = 202). These factors, which have also been noted as limitations of the PDP by the US Government Accountability Office (GAO) [63], limit our ability to quantitatively assess temporal trends in neonicotinoid residues in food commodities using statistical tests. However, to address the discontinuity in the dataset and try to overcome these limitations, we aggregated the data by 2 levels of specificity, Commodity Hybrid and Major Commodity, which allowed us to broadly evaluate neonicotinoid contamination in commodity categories and to qualitatively assess trends in detection frequencies over the time period (1999–2015).

Also as aforementioned, it appears that LODs in the PDP may not be the most sensitive, with LODs varying across the monitoring periods evaluated. For example, the LOD for imidacloprid in apple juice was reported as 0.003 ppm in 2012–2013, which was lower than the LOD range reported in 2007–2008 (0.009–0.02 ppm). Broccoli, on the other hand, had a LOD of 0.0003 ppm between 2006 and 2008 which increased to 0.01 ppm from 2013 to 2014. It is plausible that changes in analytical methods across monitoring cycles likely resulted in a variable number of detects and mean residue levels for individual neonicotinoids, masking the potential extent of neonicotinoid contamination in food commodities. Therefore, the low detection frequency across years should not be interpreted to mean that there are no neonicotinoid residues in food commodities. Given the widespread use of neonicotinoids on food commodities, further monitoring of neonicotinoids with more sensitive analytical methods may be warranted.

Lastly, the systemic nature of neonicotinoids and the timing and manner in which they are used (e.g. seed coating, foliar sprays, powder or liquid formulations) may impact detection by the PDP. For example, a study evaluating persistence of acetamiprid on chilis found varying levels of residues over 7 days depending on the formulations used (soluble powder or liquid). This same study also found residues remained in the soil after harvesting the chilis [64]. However the specific details about how neonicotinoids are applied on the sampled commodities are not monitored by the PDP. The PDP selects random commodity sampling locations to capture residue measurements that include pesticides applied during crop production and those applied after harvest (such as fungicides, growth regulators, and sprouting inhibitors) and to take into account residue degradation while food commodities are in storage [26]. Samples in the PDP may or may not accurately represent all neonicotinoid applications.

Despite these limitations, our study has several strengths worth noting. First, the PDP is the largest, most comprehensive US pesticide residue database and has the distinct advantage of containing data for imported commodities, allowing for comparisons between domestic and imported commodities. The PDP commodity sampling is based on a rigorous statistical design that ensures the data are reliable for use in exposure assessments and can be used to draw various conclusions about the US food supply. In fact, the US EPA regularly relies on PDP data, combined with food consumption information, to conduct dietary risk assessments for regulatory purposes. Second, by utilizing the PDP we were able to qualitatively characterize trends in residues of seven neonicotinoids for multiple commodity types over a 16-year period, a much longer period than previously reported in other studies [28, 30, 45]. Third, we created a commodity classification scheme to accommodate the temporal discontinuity in the PDP as well as provided a more robust analysis of neonicotinoid levels in food types. Our present study provides a thorough description of neonicotinoid trends based on PDP data available, covering domestic, imported and organic fruits, vegetables, diary, meat, grain, infant foods and domestic water supplies.

## Conclusion

To our knowledge, this is the first published analysis of trends for seven neonicotinoids in the US food supply from the earliest time point when USDA PDP began testing for neonicotinoids. Neonicotinoids are systemic pesticides, thus they cannot simply be washed off or removed by peeling before consumption of treated commodities. The highest detection frequencies (DFs) for neonicotinoids by year on all commodities were generally below 20%. Average DFs over the entire study period, 1999–2015, for domestic and imported commodities were also low at 4.5% Neonicotinoids were mainly detected in fruits and vegetables, with imidacloprid and acetamiprid residues as the more frequently detected neonicotinoids. Although the majority of commodities had neonicotinoid residue levels well below the US EPA’s tolerances, the detection of multiple neonicotinoids on fruits and vegetables in our study, including on commodities frequently consumed by children, the worldwide commercial expansion of neonicotinoids, systematic properties and environmental persistence of neonicotinoids coupled with limited information about their mixtures toxicity for developmental, neurological and possible endocrine disruption effects among humans [10, 38] underscores the need to further evaluate cumulative exposures and the potential health risks resulting from dietary intakes of neonicotinoids, especially among children and pregnant women. The implications of neonicotinoid exposures may be better understood if a national biomonitoring surveillance was in place. Currently no validated biomarkers for detecting neonicotinoids and/or their metabolites in urine or blood exist. However, the CDC’s National Center for Environmental Health Division of Laboratory Sciences (DLS) is validating analytical methods for neonicotinoids in urine. Preliminary data from DLS indicate that neonicotinoid metabolites rather than parent compounds would serve as more appropriate biomarkers since parent neonicotinoid compounds were not widely detected in urine samples [65]. The availability of exposure biomarkers for neonicotinoids will be instrumental in future studies investigating relationships between total dietary intake, and their potential health effects. Linking biomonitoring data with PDP data will also serve as important tools for not only evaluating dietary exposures but also the impact of future regulatory policies on agricultural practices of neonicotinoid applications.

## Additional files

Additional file 1: Commodities from PDP databases, import status, import fraction, and classifications, 1999–2015. (DOCX 21 kb)

Additional file 2: LODs by Major Commodity, 1999–2015 (XLSX 134 kb)

Additional file 3: Summary of Neonicotinoid Concentrations by CommodHybrid Groups, 1999 - 2015 (XLSX 45 kb)

Additional file 4: Summary of Neonicotinoid Concentrations by Major Commodity, Domestic/Import, 1999 - 2015 (DOCX 17 kb)

Additional file 5: Number of samples tested for neonicotinoids by the PDP, by year and neonicotinoid type, 1999–2015. The number of samples for acetamiprid, imidacloprid, clothianidin and thiamethoxam are similar from 2013 to 2015. (PNG 15 kb)

Additional file 6: Sensitivity Analysis of Limits of Detection (LOD) for Imidacloprid. (XLSX 11 kb)

Additional file 7: Descriptive statistics by sample type for water samples from PDP 2001–2013, by year and neonicotinoid type. All concentrations in ppt. (DOCX 15 kb)

## Abbreviations

ADI: Acceptable Daily Intake
DF: Detection frequency
EFSA: European Food Safety Agency
EU: European Union
FDA: Food and Drug Administration
FQPA: Food Quality Protection Act
GAO: Government Accountability Office
LOD: Limit of detection
nAChRs: Nicotinic acetylcholine receptors
PDP: Pesticide Data Program
US EPA: US Environmental Protection Agency
US: United States
USDA: US Department of Agriculture
