# Supplementary material for: Trends in neonicotinoid pesticide residues in food and water in the United States, 1999–2015
Source: Environ Health. 2019 Jan 11;18:7. doi: 10.1186/s12940-018-0441-7 (PMC6330495; doi:10.1186/s12940-018-0441-7)
Supplement: Supplementary file 1 — Commodities from PDP databases, import status, import fraction, and classifications, 1999–2015. (DOCX 21 kb) [file 12940_2018_441_MOESM1_ESM.docx]

| Additional File 1. Commodities from PDP databases, import status, import fraction, and classifications, 1999-2015 | | | | | | | | | | | |
| --- | --- | --- | --- | --- | --- | --- | --- | --- | --- | --- | --- |
| **Commodity** | **Imported Data Available** | **Import Fraction Average** | **Import Fraction**  **Maximum** | | | **USDA**  **Commodity**  **Code** | **Commodity - 2nd level COMMODHYBRID** | | | **Commodity - 3rd level MAJORCOMMOD** | |
| Asparagus, Canned | No | 0% | 0% | AA | | | | Asparagus | | | Vegetable |
| Black Beans, Canned | Yes | 2% | 2% | AB | | | | | Bean | | Vegetable |
| Apple Sauce | Yes | 3% | 3% | AC | | | | | Apple | | Fruit |
| Apple Juice | Yes | 47% | 60% | AJ | | | | | Apple | | Fruit |
| Almonds | Yes | 4% | 6% | AL | | | | | Almonds | | Nuts |
| Apples | Yes | 5% | 8% | AP | | | | | Apple | | Fruit |
| Asparagus | Yes | 82% | 87% | AS | | | | | Asparagus | | Vegetable |
| Apples-Single Servings | No | 0% | 0% | AX | | | | | Apple | | Fruit |
| Avocados | Yes | 74% | 74% | AV | | | | | Avocado | | Vegetable |
| Beef Adipose | Yes | 1% | 1% | BA | | | | | Meat | | Meat |
| Blueberries, Cultivated | Yes | 55% | 56% | BB | | | | | Blueberries | | Fruit |
| Beef Liver | No | 0% | 0% | BL | | | | | Meat | | Meat |
| Beef Muscle | No | 0% | 0% | BM | | | | | Meat | | Meat |
| Bananas | Yes | 100% | 100% | BN | | | | | Banana | | Fruit |
| Broccoli | Yes | 7% | 12% | BR | | | | | Broccoli | | Vegetable |
| Beets | Yes | 1% | 1% | BT | | | | | Beets | | Vegetable |
| Butter | Yes | 0% | 0% | BU | | | | | Dairy | | Dairy |
| Barley | Yes | 2% | 2% | BY | | | | | Barley | | Grain |
| Blueberries (Cultivated), Frozen | Yes | 32% | 53% | BZ | | | | | Blueberries | | Fruit |
| Cranberries | Yes | 6% | 6% | CA | | | | | Berries | | Fruit |
| Sweet Corn, Fresh | Yes | 16% | 23% | CB | | | | | Corn | | Grain |
| Peaches, Canned | No | 0% | 0% | CC | | | | | Peach | | Fruit |
| Celery | Yes | 4% | 7% | CE | | | | | Celery | | Vegetable |
| Cauliflower | Yes | 3% | 4% | CF | | | | | Cauliflower | | Vegetable |
| Cabbage | Yes | 5% | 5% | CG | | | | | Cabbage | | Vegetable |
| Cherries | Yes | 17% | 26% | CH | | | | | Cherries | | Fruit |
| Cilantro | Yes | 16% | 17% | CL | | | | | Cilantro | | Vegetable |
| Heavy Cream | No | 0% | 0% | CM | | | | | Dairy | | Dairy |
| Cantaloupe | Yes | 51% | 81% | CN | | | | | Cantaloupe | | Fruit |
| Corn Grain | No | 0% | 0% | CO | | | | | Corn | | Grain |
| Pears, Canned | No | 0% | 0% | CP | | | | | Pear | | Fruit |
| Carrots | Yes | 16% | 20% | CR | | | | | Carrot | | Vegetable |
| Sweet Corn, Frozen | Yes | 9% | 14% | CS | | | | | Corn | | Vegetable |
| Cherry Tomatoes | Yes | 48% | 52% | CT | | | | | Tomato | | Vegetable |
| Cucumbers | Yes | 54% | 59% | CU | | | | | Cucumber | | Vegetable |
| Peaches-Single Servings | No | 0% | 0% | CX | | | | | Peach | | Fruit |
| Corn Syrup | No | 0% | 0% | CY | | | | | Corn | | Grain |
| Cherries, frozen | Yes | 50% | 62% | CZ | | | | | Cherries | | Fruit |
| Infant Formula, Dairy | No | 0% | 0% | DF | | | | | Dairy | | Dairy |
| Eggs | No | 0% | 0% | EG | | | | | Egg | | Meat |
| Eggplant | No | 0% | 0% | EP | | | | | Eggplant | | Vegetable |
| Fish, Catfish | Yes | 23% | 24% | FC | | | | | Fish | | Meat |
| Fish, Salmon | Yes | 72% | 73% | FS | | | | | Fish | | Meat |
| Green Beans | Yes | 18% | 22% | GB | | | | | Green Bean | | Vegetable |
| Green Beans, Canned | Yes | 1% | 1% | GC | | | | | Green Bean | | Vegetable |
| Grapefruit | Yes | 1% | 2% | GF | | | | | Grapefruit | | Fruit |
| Grape Juice | Yes | 10% | 12% | GJ | | | | | Grape Juice | | Fruit |
| Greens, Kale | Yes | 11% | 13% | GK | | | | | Greens, Kale | | Vegetable |
| Greens, Collard | Yes | 1% | 3% | GL | | | | | Greens, Collard | | Vegetable |
| Green Onions | Yes | 78% | 82% | GO | | | | | Onion | | Vegetable |
| Grapes | Yes | 49% | 54% | GR | | | | | Grapes | | Fruit |
| Green Beans, Frozen | Yes | 10% | 10% | GZ | | | | | Green Bean | | Vegetable |
| Hot Peppers | Yes | 41% | 48% | HP | | | | | Pepper | | Vegetable |
| Honey | Yes | 34% | 37% | HY | | | | | Honey | | Meat |
| Baby Food - Carrots | Yes | 0% | 0% | IC | | | | | Carrot | | Vegetable |
| Baby Food - Peaches | Yes | 69% | 69% | IH | | | | | Peach | | Fruit |
| Baby Food - Peas | No | 0% | 0% | IE | | | | | Peas | | Vegetable |
| Baby Food - Green Beans | No | 0% | 0% | IG | | | | | Green Bean | | Vegetable |
| Baby Food - Pears | Yes | 34% | 35% | IP | | | | | Pear | | Fruit |
| Baby Food - Sweet Potatoes | No | 0% | 0% | IS | | | | | Sweet potato | | Vegetable |
| Pork Adipose | Yes | 2% | 2% | KA | | | | | Meat | | Meat |
| Kidney Beans, Canned | Yes | 3% | 4% | KB | | | | | Bean | | Vegetable |
| Pork Muscle | Yes | 2% | 2% | KM | | | | | Pork | | Meat |
| Lettuce, Organic | Yes | 18% | 18% | LO | | | | | Lettuce | | Vegetable |
| Lettuce | Yes | 2% | 4% | LT | | | | | Lettuce | | Vegetable |
| Mangoes | Yes | 97% | 97% | MA | | | | | Mango | | Fruit |
| Milk | No | 0% | 0% | MK | | | | | Dairy | | Dairy |
| Mushrooms | Yes | 12% | 13% | MU | | | | | Mushroom | | Vegetable |
| Pinto Beans, Canned | Yes | 2% | 2% | NB | | | | | Bean | | Vegetable |
| Nectarines | Yes | 25% | 42% | NE | | | | | Nectarine | | Fruit |
| Oats, Rolled/Bran | Yes | 54% | 100% | OA | | | | | Oats | | Grain |
| Oranges | Yes | 6% | 10% | OG | | | | | Citrus | | Fruit |
| Orange Juice | Yes | 44% | 88% | OJ | | | | | Oranges | | Fruit |
| Onion | Yes | 9% | 10% | ON | | | | | Onion | | Vegetable |
| Poultry Adipose | No | 0% | 0% | PA | | | | | Meat | | Meat |
| Peanut Butter | Yes | 2% | 2% | PB | | | | | Peanut butter | | Nuts |
| Peaches | Yes | 28% | 49% | PC | | | | | Peach | | Fruit |
| Plums, Dried (Prunes) | No | 0% | 0% | PD | | | | | Plum | | Fruit |
| Pears | Yes | 13% | 20% | PE | | | | | Pear | | Fruit |
| Pear Juice, Conc./Puree | Yes | 10% | 21% | PJ | | | | | Pear | | Fruit |
| Poultry Liver | No | 0% | 0% | PL | | | | | Meat | | Meat |
| Poultry Muscle | No | 0% | 0% | PM | | | | | Meat | | Meat |
| Pineapples | No | 0% | 0% | PN | | | | | Pineapples | | Fruit |
| Potatoes | Yes | 2% | 3% | PO | | | | | Potato | | Vegetable |
| Sweet Bell Peppers | Yes | 37% | 63% | PP | | | | | Pepper | | Vegetable |
| Poultry, Breast | No | 0% | 0% | PR | | | | | Meat | | Meat |
| Sweet Peas, Frozen | Yes | 6% | 12% | PS | | | | | Peas | | Vegetable |
| Poultry, Thigh | No | 0% | 0% | PT | | | | | Meat | | Meat |
| Plums | Yes | 31% | 53% | PU | | | | | Plum | | Fruit |
| Pears-Single Servings | No | 0% | 0% | PX | | | | | Pear | | Fruit |
| Potatoes, Frozen | Yes | 19% | 24% | PZ | | | | | Potato | | Vegetable |
| Raisins | Yes | 1% | 1% | RA | | | | | Raisin | | Fruit |
| Rice | Yes | 16% | 26% | RI | | | | | Rice | | Grain |
| Raspberries | Yes | 33% | 33% | RS | | | | | Berries | | Fruit |
| Raspberries, Frozen | Yes | 87% | 87% | RZ | | | | | Berries | | Fruit |
| Spinach, Canned | No | 0% | 0% | SC | | | | | Spinach | | Vegetable |
| Spinach, Frozen | Yes | 5% | 7% | SF | | | | | Spinach | | Vegetable |
| Spinach | Yes | 4% | 6% | SP | | | | | Spinach | | Vegetable |
| Summer Squash | Yes | 42% | 53% | SS | | | | | Summer Squash | | Vegetable |
| Strawberries | Yes | 10% | 16% | ST | | | | | Strawberry | | Fruit |
| Snap Peas | Yes | 70% | 70% | SN | | | | | Peas | | Vegetable |
| Sweet Potatoes | Yes | 1% | 1% | SW | | | | | Sweet potato | | Vegetable |
| Soybean grain | No | 0% | 0% | SY | | | | | Soybean | | Grain |
| Strawberries, Frozen | No | 0% | 0% | SZ | | | | | Strawberry | | Fruit |
| Tomatoes, Canned | No | 0% | 0% | TC | | | | | Tomato | | Vegetable |
| Tomatoes | Yes | 43% | 54% | TO | | | | | Tomato | | Vegetable |
| Tomato Paste | Yes | 0% | 0% | TP | | | | | Tomato | | Vegetable |
| Water, Bottled | No | 0% | 0% | WB | | | | | Water | | Water |
| Wheat Flour | Yes | 1% | 1% | | WF | | | | Wheat | | Grain |
| Water, Groundwater | No | 0% | 0% | | WG | | | | Water | | Water |
| Wheat grain | No | 0% | 0% | | WH | | | | Wheat | | Grain |
| Watermelon | Yes | 44% | 75% | | WM | | | | Watermelon | | Fruit |
| Water, Finished | No | 0% | 0% | | WR | | | | Water | | Water |
| Winter Squash | Yes | 48% | 85% | | WS | | | | Squash | | Vegetable |
| Water, Untreated | No | 0% | 0% | | WU | | | | Water | | Water |
| Winter Squash, Frozen | No | 0% | 0% | | WZ | | | | Squash | | Vegetable |
| Papaya | Yes | 77% | 79% | | YA | | | | Papaya | | Fruit |
| Infant Formula, Soy-based | Yes | 1% | 1% | | YF | | | | Soybean | | Grain |
| Garbanzo Beans, Canned | Yes | 3% | 4% | | ZB | | | | Bean | | Vegetable |

^1^The fraction imported represents the average of samples in the PDP database tested for neonicotinoids that are imported by year.
