# Supplementary material for: Trends in neonicotinoid pesticide residues in food and water in the United States, 1999–2015
Source: Environ Health. 2019 Jan 11;18:7. doi: 10.1186/s12940-018-0441-7 (PMC6330495; doi:10.1186/s12940-018-0441-7)
Supplement: Supplementary file 4 — Summary of Neonicotinoid Concentrations by Major Commodity, Domestic/Import, 1999 - 2015 (DOCX 17 kb) [file 12940_2018_441_MOESM4_ESM.docx]

Additional File 4 Summary of Neonicotinoid Concentrations by Major Commodity, Domestic and Imports

|  |  | Summary of Neonicotinoid Concentrations by Major Commodity, Domestic, 1999 - 2015 | | | | | | | Summary of Neonicotinoid Concentrations by Major Commodity,  Imports, 1999 - 2015 | | | | | | |
| --- | --- | --- | --- | --- | --- | --- | --- | --- | --- | --- | --- | --- | --- | --- | --- |
| Neonic | MajorCommod | N | N > LOD | DF% | Mean Conc (ppm) | Min Conc (ppm) | Max Conc (ppm) | Commodity with max conc (year, domestic or import and country of origin) | N | N > LOD | DF% | Mean Conc (ppm) | Min Conc (ppm) | Max Conc (ppm) | Commodity with max conc (year, domestic or import and country of origin) |
| Acetamiprid | Fruits | 22285 | 3199 | 14.35 | 0.0048097 | 0.001 | 1.5 | Raspberries(2013) | 11322 | 1298 | 11.46 | 0.0032919 | 0.001 | 0.6325 | Grapes(2015, import from Chile) |
|  | Vegetables | 39869 | 1167 | 2.93 | 0.0011413 | 0.001 | 1.6 | Greens, Kale(2007); Spinach(2009) | 9588 | 419 | 4.37 | 0.0010159 | 0.001 | 0.63 | Greens, Collard(2007, import from Mexico) |
|  | Meat | 4370 | 0 | 0 | 0 | 0 | 0 |  | 1078 | 0 | 0 | 0 | 0 | 0 |  |
|  | Dairy | 4440 | 0 | 0 | 0 | 0 | 0 |  | 3 | 0 | 0 | 0 | 0 | 0 |  |
|  | Grain (includes Rice) | 2208 | 0 | 0 | 0 | 0 | 0 |  | 315 | 0 | 0 | 0 | 0 | 0 |  |
|  | Nuts | 300 | 0 | 0 | 0 | 0 | 0 |  | 7 | 0 | 0 | 0 | 0 | 0 |  |
| Clothianidin | Fruits | 21192 | 291 | 1.37 | 0.00056094 | 0.0013 | 0.5082 | Grapes(2015) | 13406 | 42 | 0.31 | 4.3137E-05 | 0.0028 | 0.11 | Grapes(2009, import from Mexico) |
|  | Vegetables | 35403 | 744 | 2.1 | 0.00040737 | 0.0025 | 0.38 | Spinach(2015) | 8115 | 336 | 4.14 | 0.00069686 | 0.0025 | 0.25 | Cherry Tomatoes(2012, import from Mexico) |
|  | Meat | 2370 | 0 | 0 | 0 | 0 | 0 |  | 1064 | 0 | 0 | 0 | 0 | 0 |  |
|  | Dairy | 2187 | 0 | 0 | 0 | 0 | 0 |  | 1 | 0 | 0 | 0 | 0 | 0 |  |
|  | Grain (includes Rice) | 5880 | 1 | 0.02 | 4.76E-07 | 2.80E-03 | 0.0028 | Sweet Corn, Frozen(2014) | 382 | 0 | 0 | 0 | 0 | 0 |  |
|  | Nuts | 300 | 0 | 0 | 0 | 0 | 0 |  | 7 | 0 | 0 | 0 | 0 | 0 |  |
| Dinotefuran | Fruits | 17984 | 168 | 0.93 | 0.0004011 | 0.004 | 0.35 | Grapes(2009) | 10695 | 19 | 0.18 | 3.2352E-05 | 0.004 | 0.095 | Watermelon(2015, import from Mexico) |
|  | Vegetables | 31578 | 505 | 1.6 | 0.00082702 | 0.004 | 3 | Cherry Tomatoes(2012) | 8173 | 197 | 2.41 | 0.0012654 | 0.0076 | 0.81 | Sweet Bell Peppers(2011, import from Mexico) |
|  | Meat | 2214 | 0 | 0 | 0 | 0 | 0 |  | 686 | 0 | 0 | 0 | 0 | 0 |  |
|  | Dairy | 2967 | 0 | 0 | 0 | 0 | 0 |  | 3 | 0 | 0 | 0 | 0 | 0 |  |
|  | Grain (includes Rice) | 2208 | 3 | 0.14 | 5.6159E-05 | 0.037 | 0.0485 | Rice(2014) | 315 | 0 | 0 | 0 | 0 | 0 |  |
|  | Nuts | 300 | 0 | 0 | 0 | 0 | 0 |  | 7 | 0 | 0 | 0 | 0 | 0 |  |
| Flonicamid | Fruits | 16623 | 265 | 1.59 | 0.0015715 | 0.0017 | 0.6 | Strawberries(2015) | 9728 | 6 | 0.06 | 0.00005037 | 0.006 | 0.19 | Strawberries(2015, import from Mexico) |
|  | Vegetables | 27072 | 641 | 2.37 | 0.0055369 | 0.0017 | 3.8 | Spinach(2008) | 8148 | 436 | 5.35 | 0.0051776 | 0.0017 | 3.7 | Spinach(2008, import from Mexico) |
|  | Meat | 743 | 0 | 0 | 0 | 0 | 0 |  | 457 | 0 | 0 | 0 | 0 | 0 |  |
|  | Dairy | 3659 | 0 | 0 | 0 | 0 | 0 |  | 1 | 0 | 0 | 0 | 0 | 0 |  |
|  | Grain (includes Rice) | 1693 | 0 | 0 | 0 | 0 | 0 |  | 209 | 0 | 0 | 0 | 0 | 0 |  |
|  | Nuts | 0 | 0 | 0 | 0 | 0 | 0 |  | 0 | 0 | 0 | 0 | 0 | 0 |  |
| Imidacloprid | Fruits | 33782 | 2737 | 8.1 | 0.0020978 | 0.0002 | 0.59 | Grapes(2010) | 16141 | 1204 | 7.46 | 0.0068153 | 0.0002 | 2.3 | Grapes(2010, import from Chile) |
|  | Vegetables | 44880 | 9496 | 21.16 | 0.0035272 | 0.0002 | 1.5 | Broccoli(2013) | 9818 | 1406 | 14.32 | 0.0038713 | 0.0002 | 1.1 | Cilantro(2010, import from Mexico) |
|  | Meat | 4378 | 0 | 0 | 0 | 0 | 0 |  | 1100 | 0 | 0 | 0 | 0 | 0 |  |
|  | Dairy | 4440 | 1 | 0.02 | 1.19E-06 | 5.30E-03 | 0.0053 | Butter(2012) | 3 | 0 | 0 | 0 | 0 | 0 |  |
|  | Grain (includes Rice) | 7307 | 0 | 0 | 0 | 0 | 0 |  | 767 | 1 | 0.13 | 1.4342E-05 | 0.011 | 0.011 | Rice(2009, import from India) |
|  | Nuts | 824 | 0 | 0 | 0 | 0 | 0 |  | 28 | 0 | 0 | 0 | 0 | 0 |  |
| Thiacloprid | Fruits | 19438 | 452 | 2.33 | 0.00017819 | 0.0007 | 0.13 | Apples(2010) | 10156 | 451 | 4.44 | 0.0012932 | 0.001 | 0.3385 | Pears(2015, import from Chile) |
|  | Vegetables | 19911 | 3 | 0.02 | 7.78E-07 | 2.00E-03 | 0.011 | Hot Peppers(2010) | 5944 | 56 | 0.94 | 0.00021888 | 0.0007 | 0.49 | Snap Peas (2012, import from Guatemala) |
|  | Meat | 2338 | 0 | 0 | 0 | 0 | 0 |  | 1009 | 0 | 0 | 0 | 0 | 0 |  |
|  | Dairy | 2186 | 0 | 0 | 0 | 0 | 0 |  | 1 | 0 | 0 | 0 | 0 | 0 |  |
|  | Grain (includes Rice) | 1372 | 0 | 0 | 0 | 0 | 0 |  | 190 | 0 | 0 | 0 | 0 | 0 |  |
|  | Nuts | 0 | 0 | 0 | 0 | 0 | 0 |  | 0 | 0 | 0 | 0 | 0 | 0 |  |
| Thiamethoxam | Fruits | 24763 | 554 | 2.24 | 0.00056473 | 0.0013 | 0.25 | Strawberries(2009) | 14440 | 173 | 1.2 | 0.00018288 | 0.002 | 0.28 | Tangerines (2012, import from South Africa) |
|  | Vegetables | 45469 | 1128 | 2.48 | 0.00031089 | 0.002 | 0.36 | Summer Squash(2013) | 10631 | 927 | 8.72 | 0.001967 | 0.002 | 0.38 | Cherry Tomatoes(2012, import from Mexico) |
|  | Meat | 2723 | 0 | 0 | 0 | 0 | 0 |  | 1093 | 0 | 0 | 0 | 0 | 0 |  |
|  | Dairy | 3709 | 0 | 0 | 0 | 0 | 0 |  | 3 | 0 | 0 | 0 | 0 | 0 |  |
|  | Grain (includes Rice) | 6870 | 0 | 0 | 0 | 0 | 0 |  | 393 | 1 | 0.25 | 6.36E-06 | 2.50E-03 | 0.0025 | Sweet Corn, Fresh(2010, import from Mexico) |
|  | Nuts | 300 | 0 | 0 | 0 | 0 | 0 |  | 7 | 0 | 0 | 0 | 0 | 0 |  |

*just among samples above LOD; See Additional File 2 for LODs by Major Commodity, 1999 - 2015
