# Supplementary figures and images for: Trends in neonicotinoid pesticide residues in food and water in the United States, 1999–2015
Source: Environ Health. 2019 Jan 11;18:7. doi: 10.1186/s12940-018-0441-7 (PMC6330495; doi:10.1186/s12940-018-0441-7)

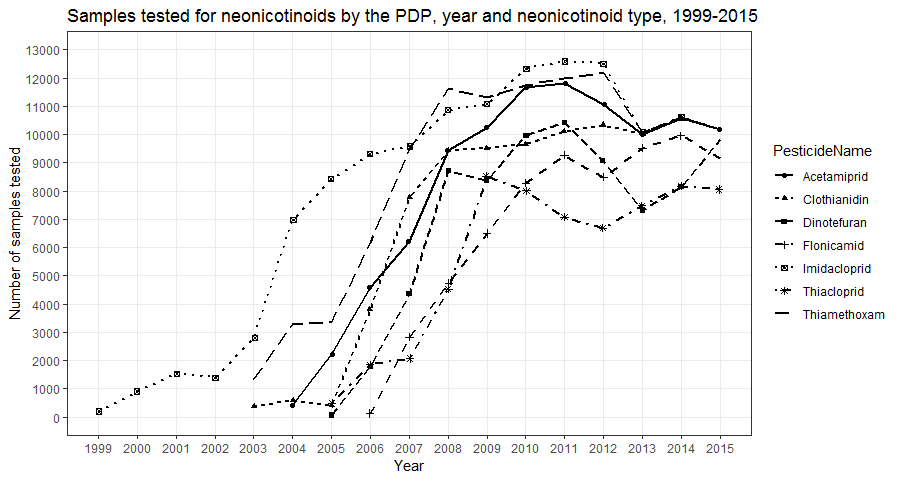

Supplement: Supplementary file 5 — Number of samples tested for neonicotinoids by the PDP, by year and neonicotinoid type, 1999–2015. The number of samples for acetamiprid, imidacloprid, clothianidin and thiamethoxam are similar from 2013 to 2015. (PNG 15 kb) [file 12940_2018_441_MOESM5_ESM.png]
