# Supplementary material for: Trends in neonicotinoid pesticide residues in food and water in the United States, 1999–2015
Source: Environ Health. 2019 Jan 11;18:7. doi: 10.1186/s12940-018-0441-7 (PMC6330495; doi:10.1186/s12940-018-0441-7)
Supplement: Supplementary file 7 — Descriptive statistics by sample type for water samples from PDP 2001–2013, by year and neonicotinoid type. All concentrations in ppt. (DOCX 15 kb) [file 12940_2018_441_MOESM7_ESM.docx]

Additional File 7 Descriptive statistics by sample type for water samples+ from 2001-2013, by year and neonicotinoid type

|  |  | Groundwater | | | | | Water, finished | | | | | Water, untreated | | | | |
| --- | --- | --- | --- | --- | --- | --- | --- | --- | --- | --- | --- | --- | --- | --- | --- | --- |
|  |  | Number | Mean* | Conc. Range* | | % positive | Number | Mean* | Conc. Range* | | % positive | Number | Mean* | Conc. Range* | | % positive |
| Pesticide Name | Year |  |  | Min | Max |  |  |  | Min | Max |  |  |  | Min | Max |  |
| Acetamiprid | 2010 | 228 | 0 | 0 | 0 | 0.0 | 189 | 0 | 0 | 0 | 0.0 | 189 | 0 | 0 | 0 | 0.0 |
|  | 2011 | 606 | 0 | 0 | 0 | 0.0 | 0 | . | . | . | . | 0 | . | . | . | . |
|  | 2012 | 166 | 0 | 0 | 0 | 0.0 | 232 | 0 | 0 | 0 | 0.0 | 253 | 0 | 0 | 0 | 0.0 |
|  | 2013 | 14 | 0 | 0 | 0 | 0.0 | 50 | 0 | 0 | 0 | 0.0 | 50 | 0 | 0 | 0 | 0.0 |
| Clothianidin | 2010 | 0 | . | . | . | . | 189 | 0.25 | 0 | 8 | 3.2 | 189 | 0.25 | 0 | 8 | 3.2 |
|  | 2011 | 606 | 0 | 0 | 0 | 0.0 | 0 | . | . | . | . | 0 | . | . | . | . |
|  | 2012 | 166 | 0 | 0 | 0 | 0.0 | 232 | 0.26 | 0 | 12.50 | 3.0 | 253 | 0.74 | 0 | 29 | 6.3 |
|  | 2013 | 14 | 0 | 0 | 0 | 0.0 | 50 | 0.68 | 0 | 18 | 6.0 | 50 | 1.84 | 0 | 45 | 8.0 |
| Dinotefuran | 2012 | 101 | 0 | 0 | 0 | 0.0 | 54 | 0 | 0 | 0 | 0.0 | 54 | 0.23 | 0 | 12.5 | 1.9 |
|  | 2013 | 12 | 0 | 0 | 0 | 0.0 | 7 | 0 | 0 | 0 | 0.0 | 7 | 0 | 0 | 0 | 0.0 |
| Imidacloprid | 2001 | 0 | . | . | . | . | 100 | 0 | 0 | 0 | 0.0 | 0 | . | . | . | . |
|  | 2002 | 0 | . | . | . | . | 281 | 0.01 | 0 | 2.50 | 0.4 | 0 | . | . | . | . |
|  | 2003 | 0 | . | . | . | . | 583 | 0.01 | 0 | 2.50 | 0.3 | 0 | . | . | . | . |
|  | 2004 | 0 | . | . | . | . | 238 | 0.06 | 0 | 2.50 | 2.5 | 238 | 0.04 | 0 | 2.5 | 1.7 |
|  | 2005 | 0 | . | . | . | . | 230 | 0 | 0 | 0 | 0.0 | 231 | 0 | 0 | 0 | 0.0 |
|  | 2006 | 0 | . | . | . | . | 365 | 0.11 | 0 | 5.5 | 4.1 | 367 | 0.70 | 0 | 202 | 5.2 |
|  | 2007 | 0 | . | . | . | . | 368 | 0.07 | 0 | 2.5 | 3.0 | 362 | 0.09 | 0 | 2.5 | 3.6 |
|  | 2008 | 0 | . | . | . | . | 309 | 0.43 | 0 | 12 | 12.9 | 308 | 2.54 | 0 | 100 | 17.2 |
|  | 2009 | 0 | . | . | . | . | 306 | 0.29 | 0 | 21 | 7.2 | 305 | 1.45 | 0 | 29 | 17.0 |
|  | 2010 | 242 | 0.86 | 0 | 124 | 2.5 | 284 | 1.55 | 0 | 67 | 11.3 | 282 | 1.69 | 0 | 60 | 14.9 |
|  | 2011 | 606 | 0.24 | 0 | 37 | 1.5 | 118 | 2.96 | 0 | 99 | 29.7 | 120 | 3.51 | 0 | 77 | 36.7 |
|  | 2012 | 166 | 0.33 | 0 | 13 | 3.6 | 232 | 0.31 | 0 | 6 | 5.2 | 253 | 0.49 | 0 | 14 | 6.7 |
|  | 2013 | 14 | 0 | 0 | 0 | 0.0 | 50 | 0 | 0 | 0 | 0.0 | 50 | 0 | 0 | 0 | 0.0 |
| Thiamethoxam | 2010 | 228 | 0 | 0 | 0 | 0.0 | 189 | 0.38 | 0 | 10.20 | 3.7 | 189 | 0.46 | 0 | 25 | 3.7 |
|  | 2011 | 606 | 0 | 0 | 0 | 0.0 | 0 | . | . | . | . | 0 | . | . | . | . |
|  | 2012 | 166 | 0 | 0 | 0 | 0.0 | 232 | 0.04 | 0 | 10.20 | 0.4 | 253 | 0.24 | 0 | 10.2 | 2.4 |
|  | 2013 | 14 | 0 | 0 | 0 | 0.0 | 50 | 0.20 | 0 | 10.20 | 2.0 | 50 | 0 | 0 | 0 | 0.0 |

+No bottled water was tested for neonicotinoids.

See Additional File 2 for all LODs

* All concentrations in ppt
